# Increased expression of the mitochondrial derived peptide, MOTS-c, in skeletal muscle of healthy aging men is associated with myofiber composition

**DOI:** 10.18632/aging.102944

**Published:** 2020-03-17

**Authors:** Randall F. D’Souza, Jonathan S. T. Woodhead, Christopher P. Hedges, Nina Zeng, Junxiang Wan, Hiroshi Kumagai, Changhan Lee, Pinchas Cohen, David Cameron-Smith, Cameron J. Mitchell, Troy L. Merry

**Affiliations:** 1 Discipline of Nutrition, Faculty of Medical and Health Sciences, The University of Auckland, Auckland, New Zealand; 2 Maurice Wilkins Centre for Molecular Biodiscovery, The University of Auckland, Auckland, New Zealand; 3 Liggins Institute, The University of Auckland, Auckland, New Zealand; 4 Department of Physiology, Faculty of Medical and Health Sciences, The University of Auckland, Auckland, New Zealand; 5 Leonard Davis School of Gerontology, University of Southern California, Los Angeles, CA 90089, USA; 6 Japan Society for the Promotion of Science, Tokyo, Japan; 7 Graduate School of Health and Sports Science, Juntendo University, Chiba, Japan; 8 USC Norris Comprehensive Cancer Center, Los Angeles, CA 90033, USA; 9 Biomedical Science, Graduate School, Ajou University, Suwon, Korea; 10 School of Kinesiology, University of British Colombia, Vancouver, BC V6T 1Z1, Canada

**Keywords:** muscle, mitochondria, mitochondrial derived peptides, aging, MOTS-c

## Abstract

Mitochondria putatively regulate the aging process, in part, through the small regulatory peptide, mitochondrial open reading frame of the 12S rRNA-c (MOTS-c) that is encoded by the mitochondrial genome. Here we investigated the regulation of MOTS-c in the plasma and skeletal muscle of healthy aging men. Circulating MOTS-c reduced with age, but older (70-81 y) and middle-aged (45-55 y) men had ~1.5-fold higher skeletal muscle MOTS-c expression than young (18-30 y). Plasma MOTS-c levels only correlated with plasma in young men, was associated with markers of slow-type muscle, and associated with improved muscle quality in the older group (maximal leg-press load relative to thigh cross-sectional area). Using small mRNA assays we provide evidence that MOTS-c transcription may be regulated independently of the full length 12S rRNA gene in which it is encoded, and expression is not associated with antioxidant response element (ARE)-related genes as previously seen in culture. Our results suggest that plasma and muscle MOTS-c are differentially regulated with aging, and the increase in muscle MOTS-c expression with age is consistent with fast-to-slow type muscle fiber transition. Further research is required to determine the molecular targets of endogenous MOTS-c in human muscle but they may relate to factors that maintain muscle quality.

## INTRODUCTION

Aging is characterized by a progressive decline in physiological function [1], which is controlled by a complex interaction between environmental and genetic factors [2]. While understanding the biological mechanisms of aging that result from these interactions is an area of intensive investigation, building evidence suggests that alterations in mitochondrial function plays a central role in coordinating the aging process [3]. Mitochondrial bioenergetics of skeletal muscle decline with age, and this decrement may pre-dispose to certain age-related diseases [4–6]. However, the mitochondrial biology of aging theory extends beyond that of altered intrinsic efficiencies in energy production to include a control over inflammatory responses, proteostasis, oxidative balance, stem cell function and initiation of adaptive stress responses [3, 7–9]. Coordination of such a complex array of cellular processes requires diverse mitochondrial initiated pathways of intra- and extra-cellular communication [10–12].

The mammalian mitochondrial proteome consists of >1000 proteins, of which 99% are encoded by the nuclear genome. All 13 proteins traditionally recognized as being encoded by mitochondrial DNA (mtDNA) form essential components of the oxidative phosphorylation (OXPHOS) complexes, with no known nuclear or mitochondrial-independent functions [13]. However, short open reading frames (sORFs) within the mitochondrial genome have recently been identified as harboring sequences for small regulatory peptides [14–17]. These mitochondrial-derived peptides (MDP), or mitokines, appear to form a critical retrograde communication pathway between the mitochondria and the wider cell, and may have an endocrine cytoprotective role [18–21].

MOTS-c, an MDP with its sequence located within the coding region for mitochondrial 12S rRNA gene [14], has recently been shown to be induced by metabolic perturbation and translocate to the nucleus where it is involved in regulating nuclear gene expression, including those with antioxidant response elements (ARE) to protect against metabolic stress [22, 23]. A stress mediated mitonuclear communication role of MOTS-c may partly explain why exogenous MOTS-c is capable of preventing diet, aging and menopause associated metabolic discourse and insulin resistance, but has limited impact on the resting metabolism of healthy young mice [14, 24–27]. Insulin sensitive tissues, such as skeletal muscle and fat, appear to be key target sites of MOTS-c, and levels of MOTS-c in skeletal muscle and plasma of aged mice are reduced [14, 26–28]. This has led to speculation that MOTS-c is an age related mitokine [29, 30], however whether human plasma and muscle MOTS-c levels are influenced by healthy aging is unclear. Therefore, we investigated plasma and vastus lateralis muscle MOTS-c levels in human males.

We report that while plasma MOTS-c levels are reduced with age, muscle levels are increased and this may be associated with aging-related fast-to-slow fiber type transition and the reduced ability of MOTS-c to leave the cell. Furthermore, we provide evidence that MOTS-c transcription is regulated independent of 12S rRNA, and MOTS-c protein or transcript expression does not correlate well with ARE-related skeletal muscle gene expression in resting skeletal muscle during aging.

## RESULTS

### Plasma MOTS-c levels are reduced with aging while muscle levels are increased

**Figure 1:**
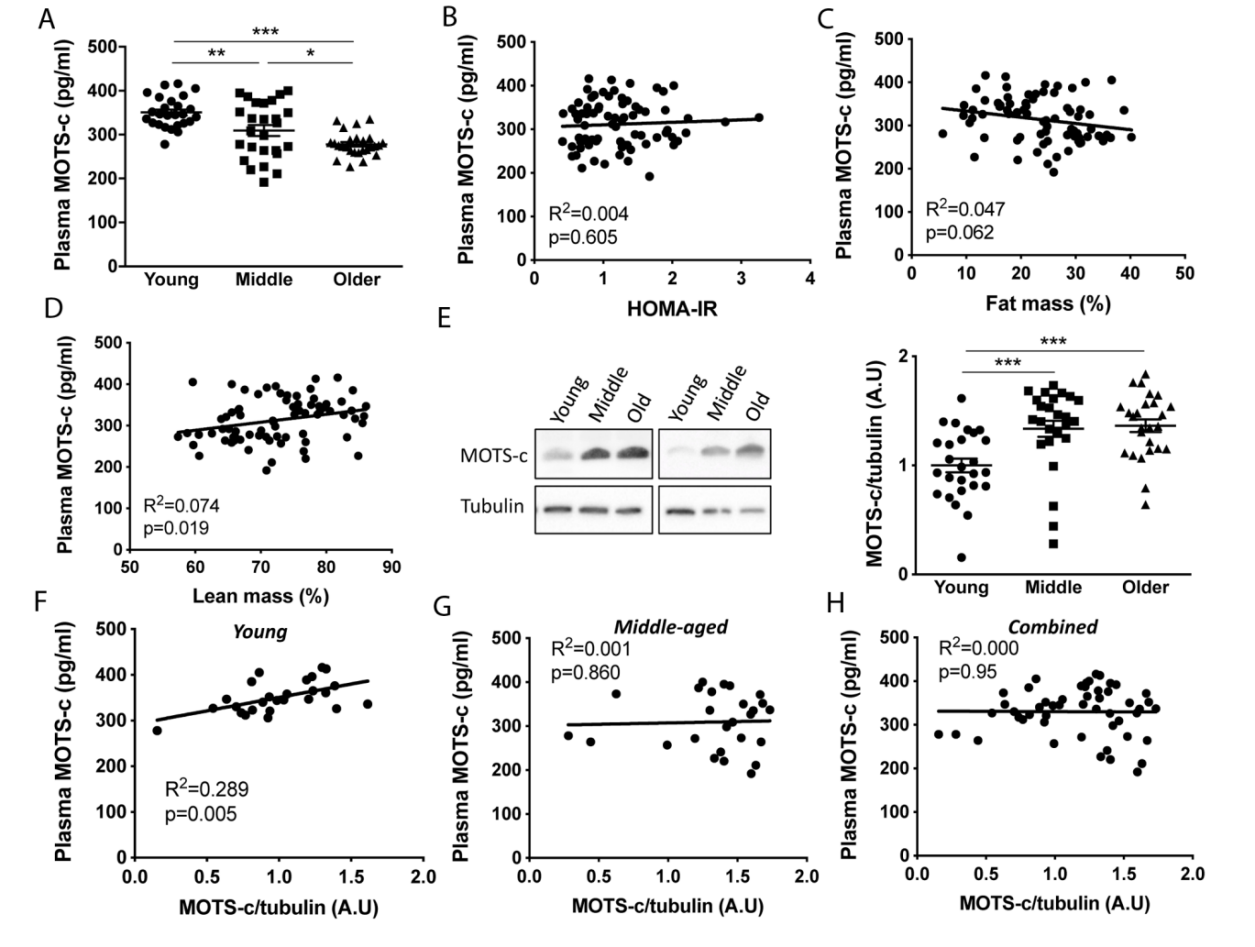
**Plasma MOTS-c levels decrease and skeletal muscle levels increase with aging.** Plasma MOTS-c (**A**), correlated with HOMA-IR (**B**), fat mass (**C**), lean mass (**D**), and muscle MOTS-c expression (**E**) in young, middle-aged and older males. Representative blots are independent and from different participants. Correlation between plasma MOTS-c and muscle MOTS-c expression in young (**F**), middle (**G**), and combined (**H**). Significance was determined using linear regression or one-way ANOVA. Data is presented as means ± SE, n=26 per group except for body composition measurements where data was not available for a young (n=25) and middle-aged (n=25) participant. *p<0.05, **p<0.01, ***p<0.001.

Plasma MOTS-c levels were measured in young (18-30 y), middle-aged (45-55 y) and older (70-81 y) males that were free from any overt disease following an overnight fast (Figure 1). Characteristics of participants are shown in Table 1, while all age groups had similar HOMA-IR, plasma LDL and HDL, the older-aged groups had higher fat mass and lower lean mass, and the middle-aged group high plasma triglycerides. Consistent with murine data [14], both the middle and older groups had lower circulating MOTS-c (by 11 and 21%, respectively) than the young group. Since exogenous MOTS-c treatment of aged or high fat diet challenged mice improves insulin sensitivity and alters body composition [14] we correlated plasma MOTS-c levels with HOMA-IR, and relative fat and lean mass (Figure 1B–1D). A weak association was observed with relative lean mass, but not fat mass or HOMA-IR (Figure 1B, 1C). To determine if differences between the groups could be explained by differences in clinical blood parameter or body composition, ANCOVA analysis was undertaken with % fat, % lean mass, HOMA-IR and plasma triglycerides as covariates (both independently and combined; Supplementary Table 1). With these covariates included the main effect for age on plasma MOTS-c remained significant (p<0.001; Supplementary Table 1) indicating that this is likely an age-dependent effect. To determine if skeletal muscle MOTS-c levels were similarly reduced with aging, MOTS-c protein expression was measured in vastus lateralis muscle samples at rest. In contrast to both plasma data, and MOTS-c muscle levels reported in aged mice [14], both the older and middle-aged groups had higher levels of muscle MOTS-c than the young group (Figure 1E and Supplementary Table 2). Plasma and muscle MOTS-c positively correlated in young, but not middle-aged or combined groups (Figure 1F–1H). The muscle and plasma samples for the older group were from different participants and therefore this comparison could not be made for the older men.

**Table 1:** Participant characteristics.

	**Young**	**Middle-aged**	**Older 1**	**Older 2**
Age (y)	22 ± 3	50 ± 2^**^	70 ± 4^**,††^	74 ± 3^**,††,‡‡^
Weight (kg)	77.8 ± 10.8	83.9 ± 9.8	88.5 ± 13.7^*^	85.7 ± 11.7
BMI (kg/m^2^)	24 ± 3	26 ± 38	28 ± 4^**^	28 ± 4^**^
Fat mass (%)	18.8 ± 7.8	23.9 ± 6.1^*^	NC	28.4 ± 4.8^**,†^
Lean mass (%)	76.9 ± 7.7	72.9 ± 5.8	NC	67.6 ± 4.6^**,††^
HOMA-IR	1.28 ± 0.67	1.11 ± 0.51	NC	1.31 ± 0.56
HDL (mmol/L)	1.35 ± 0.28	1.19 ± 0.28	NC	1.30 ± 0.34
LDL (mmol/L)	2.8 ± 0.74	3.2 ± 0.94	NC	3.01 ± 0.94
TRIG (mmol/L)	1.02 ± 0.50	1.45 ± 0.60^*^	NC	1.19 ± 0.62

Data are mean ± standard deviation for n=26 per group except for body composition measurements where data was not available for a young (n=25) and middle-aged (n=25) participant. NC=not collected; BMI= body mass index; HDL= high density lipoprotein; LDL=low density lipoprotein; TRIG= triglycerides; ^*^p<0.05 vs Young, ^**^p<0.001 vs Young, ^†^p<0.05 vs Middle-aged, ^††^p<0.01 vs Middle-aged, ^‡‡^p<0.01 vs Older 1.

### Age-related increases in muscle MOTS-c levels are associated with fast-to-slow fiber type shift

**Figure 2:**
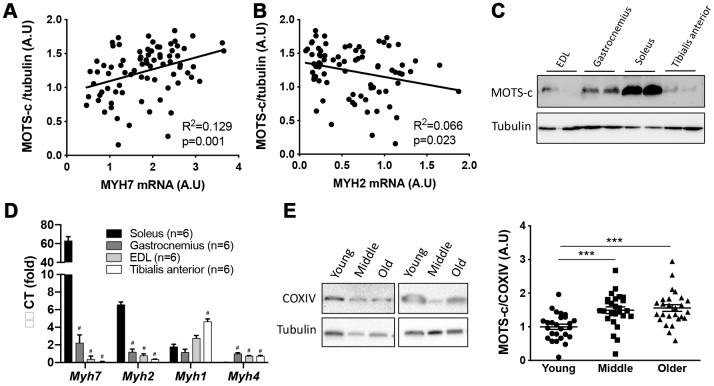
**MOTS-c expression is higher in slow-type muscle.** Correlations between muscle MOTS-c expression and *MYH7* (**A**) and *MYH2* (**B**) mRNA levels in young, middle-aged and older men. Mouse extensor digitorum longus (EDL), gastrocnemius, tibialis anterior (TA) and soleus (SOL) muscle MOTS-c expression (**C**), and mRNA levels of fiber type markers (**D**). Two independent COXIV representative blots with different participants and quantification of MOTS-c relative to COXIV expression (**E**) in muscle samples from young, middle-aged and older males. Significance was determined using linear regression or one-way ANOVA. Data is presented as means ± SE for n=26 per group. ***p<0.001; ^#^p<0.0001 vs soleus muscle.

In addition to loss of muscle mass, aging is associated with a fast-to-slow fiber type shift [31]. Therefore, we determined whether markers of slow (myosin heavy chain type 7, *MYH7*) and fast (myosin heavy chain type 2, *MYH2*) type fibers associate with muscle MOTS-c levels. Consistent with the hypothesis that a change in fiber type may account for the increase in muscle MOTS-c levels observed with aging, *MYH7* mRNA showed a positive association with muscle MOTS-c levels while *MYH2* mRNA was negatively associated (Figure 2A, 2B). Furthermore, MOTS-c expression was higher in mouse soleus muscle (Figure 2C) which has a higher proportion of slow type fibers than EDL, gastrocnemius, and tibialis anterior muscles (Figure 2C). Higher slow-type fiber content of soleus muscle was confirmed by measuring mRNA levels of Myh7 (type I fiber), Myh2 (type IIa fibers), Myh4 (type IIb fibers) and Myh1 (type IIx fibers) in these muscles (Figure 2D). Slow type fibers normally have a greater mitochondrial density, therefore the mitochondrial protein COXIV was determined in muscle samples and used to correct MOTS-c levels for mitochondrial mass. This did not change the increase in muscle MOTS-c expression observed in the middle-aged and older groups compared to the young group (Figure 2E), suggesting that the increase in muscle MOTS-c levels was independent of mitochondrial protein levels.

**Figure 3:**
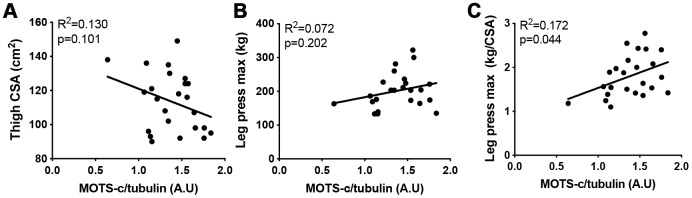
**The association between MOTS-c and muscle area and function.** Thigh cross-sectional area (CSA) (**A**), maximal leg press load (**B**) and maximal leg press load relative to CSA (**C**) was correlated with muscle MOTS-c expression in older men. Significance was determined using linear regression or one-way ANOVA. Due to missing pQCT/leg press data n=24.

To assess how muscle MOTS-c levels relate to muscle function, thigh cross-sectional area (CSA) and maximal leg press weight was measured in the older group. There was no association between MOTS-c levels and leg press weight or CSA. However, when maximal leg press weight was corrected for thigh size (CSA) a positive association was seen (Figure 3). This may suggest that muscle MOTS-c levels are associated with improved muscle quality in the elderly.

### Transcriptional regulation of MOTS-c and MT-RNR1 with age

**Figure 4:**
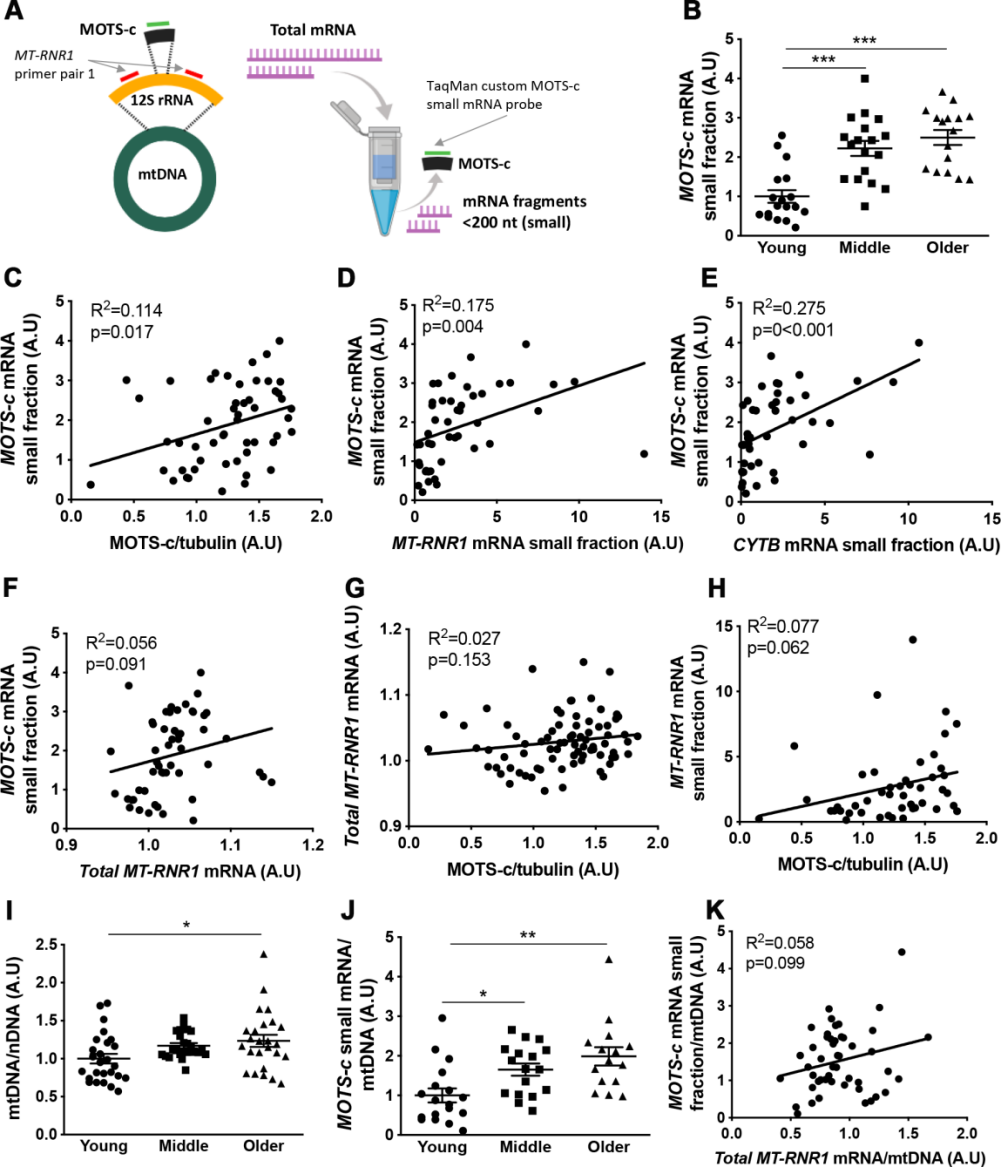
**Muscle MOTS-c and *12S rRNA* transcription with aging.** *12S rRNA mRNA* (*MT-RNR1*) and *MOTS-c* mRNA analysis technique (**A**). Young, middle-aged and older male muscle *MOTS-c* mRNA levels in the small RNA fraction were determined (**B**) and correlated with muscle MOTS-c protein expression (**C**), *MT-RNR1* (**D**) and *CYTB* (**E**) mRNA levels in the small RNA fraction and *MT-RNR1* mRNA levels in the total RNA fraction (**F**). *MT-RNR1* mRNA levels in the total (**G**) and small (**H**) RNA fraction were correlated with MOTS-c protein expression. Muscle mitochondrial to nuclear DNA (mtDNA/nDNA) (**I**), and *MOTS-c* mRNA levels relative to mtDNA (**J**), and correlation of *MOTS-c* mRNA and *MT-RNR1* mRNA in the total RNA relative to mtDNA (**K**). Significance was determined using linear regression or one-way ANOVA. Results are shown as means ± SE, due to limited sample availability and assay failure, for small RNA fraction assays n=14-18 per group, and for mtDNA n=26 for young, 24 for middle and 25 for old. *p<0.05, **p<0.01, ***p<0.001.

The coding sequence for MOTS-c is found within the *12S rRNA* gene (*MT-RNR1*) of mtDNA (Figure 4A). To determine if *MOTS-c* transcripts are present in muscle independent of *MT-RNR1* transcripts we filtered total RNA to enrich small mRNA fragments (<200 nt) and used a custom TaqMan small RNA assay with a probe that was designed against the 51-nucleotide sequence of MOTS-c to measure *MOTS-c* mRNAs in the enriched small RNA fraction. Similar to muscle MOTS-c protein expression, both the middle and older aged groups had increased muscle *MOTS-c* mRNA compared to the young group (Figure 4B), and MOTS-c mRNA correlated with MOTS-c protein levels (Figure 4C). Due to the amount of RNA required for this approach, only 52 samples could be included in this analysis; 18 each from young and middle-aged, and 16 from the older groups.

*MOTS-c* mRNA correlated with *MT-RNR1* and *CYTB* measured in the small RNA fraction but not in total RNA (Figure 4D–4F; and Supplementary Figure 1). Similar results were seen for a second primer pair designed against different location in the *MT-RNR1* gene (Supplementary Figure 1), and as a result of enriching for transcripts <200 nt, the expression of these mRNA’s was substantially lower in the small fraction compared to fractions containing larger transcripts (Supplementary Figure 2). This indicates that small RNA transcript levels are independent of the larger transcript levels, suggesting that they may be independently transcribed. Neither *MT-RNR1* mRNA measured in total or small RNA fraction correlated with muscle protein MOTS-c protein levels (Figure 4G, 4H).

To determine whether the increase in *MOTS-c* mRNA is a function of differences in mtDNA content, mitochondrial-to-nuclear ratio (mtDNA/nDNA) was measured and used to standardize *MOTS-c* mRNA levels (Figure 4I, 4J). Similar to MOTS-c protein expression, when corrected for mtDNA content, both the middle and older groups had greater *MOTS-c* mRNA relative to mtDNA than the young group (Figure 4J), and this standardization did not improve the association between *MOTS-c* mRNA and *MT-RNR1* mRNA total RNA fraction (Figure 4K).

**Figure 5:**
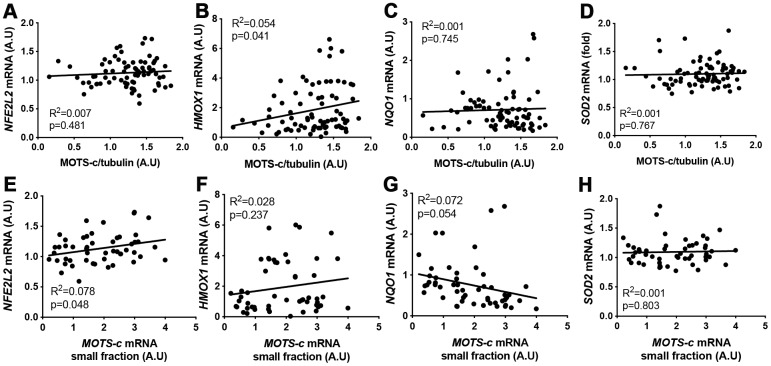
**MOTS-c muscle protein or small mRNA expression does not correlate well with antioxidant-response element (ARE) related genes.** Correlation between MOTS-c protein (**A**–**D**) or small mRNA (**E**–**H**) expression and mRNA levels of *NFE2L2*, *HMOX1*, *NQO1* and *SOD2* in muscle samples from young, middle-aged and older men. Significance was determined using linear regression for n=78 (protein correlations) or n=52 (*MOTS-c* mRNA correlations).

MOTS-c has been shown to bind to nuclear DNA in HEK293 cells and interact with Nrf2 (*NFE2L2*) to regulate ARE-related gene expression [22]. A weak correlation was observed between skeletal muscle *NRF2* mRNA and MOTS-c small mRNA levels, and *HMOX-1* mRNA and MOTS-c protein expression (Figure 5B and 5E). However, overall there was no association between ARE-responsive genes (*HMOX-1*, *NQO1* and *SOD2*) and *MOTS-c* small mRNA, or *NQO1* and *SOD2* mRNA and MOTS-c protein levels in skeletal muscle (Figure 5A–5H).

## DISCUSSION

The mitochondrial encoded peptide, MOTS-c, has been implicated in regulating the aging process from observations in murine studies [29, 30]. Here, we provide evidence that plasma MOTS-c levels are lower, but skeletal muscle levels are higher in aged human males independent of body composition, HOMA-IR and plasma triglycerides. As such, and because our participants were free from overt aging-related co-morbidities, these changes in MOTS-c expression are likely to be largely independent of aged-associated disease, fat mass, and insulin sensitivity, suggesting that changes in MOTS-c levels are the results of normal aging rather than an artifact of disease development.

While MOTS-c serum levels have recently been reported to negatively correlate with age in a cohort of participants with type 2 diabetes [32], interpretation of this data is confounded by the healthy control group being significantly younger than the diabetic groups. Regardless, our results support this finding and previous observations of lower MOTS-c plasma levels in aged mice, but are in contrast to reported murine skeletal muscle age-associated decline in MOTS-c levels [14]. This may reflect inherent differences between human and mouse skeletal muscle aging [33, 34], but also could be the result of the relative age of the mice (32 months which equates to >80 human years [35]) being older than that of human participants in this study. The middle-aged and older participants were healthy, independent and mobile, and therefore it is possible that the observed increase in muscle MOTS-c in these groups is a compensatory response to age-related muscle metabolic stress [36] aimed at coordinating pathways to maintaining muscle quality and function. This would be consistent with the recent report that metabolic and oxidative stressors increase MOTS-c expression to regulate an adaptive transcriptional response [22], and supports our observation of a positive association between muscle quality and MOTS-c muscle expression. However, when the ability of the muscle cell to tolerate or adapt to this stress is overwhelmed then MOTS-c levels may decrease along with function of the cell. In support, some studies have reported reduced plasma levels of MOTS-c in people with obesity, type 2 diabetes [32, 37] or impaired coronary endothelial function [38], and exogenous MOTS-c treatment restores skeletal muscle insulin sensitivity in mice [14, 27].

Plasma and muscle MOTS-c levels show opposing responses to aging, perhaps suggesting that at rest in a fasted state the primary source of plasma MOTS-c is not skeletal muscle or the pharmacokinetics of MOTS-c changes with age. However, interpretation of this result is limited by plasma and muscle samples from the older men being from different participants. Nevertheless, mice express MOTS-c in a wide range of tissues [25], being particularly highly expressed in the liver, an organ that is sensitive to age-related decline in function. A number of hepatokines are now suggested as being age- and metabolic-sensitive [39–41], and MOTS-c may fall into this category. Interestingly, however, we observed a strong correlation between plasma and skeletal muscle MOTS-c in young but not middle-aged groups. An intriguing alternative hypothesis is that the ability for MOTS-c to leave the muscle cell is lost with age, and this could be the result of either the loss of an export process and/or a greater need for mitochondrial retrograde signaling in aging muscle. It is also tempting to further speculate that this inability to release MOTS-c is tied to the greater expression of MOTS-c in slow-type fibers and age-related fast-to-slow fiber type transition. Indeed, fiber-type specific regulation of myokines is common with brain-derived neurotrophic factor (BDNF) promoting glycolytic fiber type transition [42] and myonectin being more highly expressed in oxidative muscle fibers [43]. Further work is required to determine whether MOTS-c has fiber-type specific roles and to identify the molecular targets of MOTS-c in human skeletal muscle. Our analysis showed little association between previously reported MOTS-c transcriptional targets and either MOTS-c protein or mRNA expression suggesting targets may differ in humans *in vivo*, or with aging. However, recently published plasma metabolomic data from MOTS-c treated obese mice suggest the lipid metabolism pathway as a key target [24].

Mitochondrial derived peptides are transcribed from sORF within mitochondrial genes and hypothesized to be translated in the cytoplasm [14]. Whether MOTS-c is translated as part of 12S rRNA transcript or independently is not known. Using a small RNA probe against the MOTS-c sequence and filtering total RNA to enrich short RNA fragments (<200 nt; lowering full length 12S rRNA mRNA) we provide evidence that MOTS-c transcripts may be differentially regulated from that of the full parent gene sequence, but similar to that of other small transcripts from the mitochondrial genome. This could be the result of sequence specific transcription and export, an artifact of lower MOTS-c mRNA stability compared to full length 12S mRNA, or reflect the formation of smaller functional mRNA transcripts during the multiple steps of mitochondrial RNA processing and export [44]. Whether the latter of these options is responsive to mitochondrial perturbations and could be a mechanism underpinning MOTS-c sensitivity to metabolic stress is yet to be determined, along with the full sequence of the MOTS-c transcript.

Taken together, our data indicate that the mitochondrial derived peptide, MOTS-c, is differentially regulated in the skeletal muscle and plasma of healthy (free of serious comorbidities) males during normal aging. We suggest that an aging-associated fast-to-slow type muscle transition may underpin the increase in muscle MOTS-c seen during aging and that this may be related to the loss of positive association between plasma and muscle in older age.

## MATERIALS AND METHODS

### Participants

One hundred and four healthy young, middle-aged or older males were recruited (Table 1 and [45]) and gave written informed consent before the commencement of the study. Participants were sedentary to recreationally active, non-smokers, free of chronic illnesses including cardiovascular or metabolic disease and were not taking any medication. Body composition was assessed by dual X-ray absorptiometry (DXA; model iDXA, GE-Lunar, Madison, WI), and thigh muscle cross-sectional area was determined via anthropometric measurement as previously reported [46]. One repetition maximum (1-RM) for leg press was estimated using the Brzycki equation [47]. Two groups of older participants were recruited as blood samples were not available for the group that had muscle samples collected (Older 1). All experimental procedures were approved by the Health and Disability (New Zealand) and Deakin University Human (Melbourne, Australia) Research Ethics Committee’s, and performed in accordance with the Helsinki declaration.

### Murine housing

Mice were maintained in a temperature-controlled animal facility with 12 h light-dark cycle and *ad libitum* access to water, a standard rodent chow diet (Teklad TB 2018; Harlan, Madison, WI). At 10-12 weeks of age in-house bred male C57BL/6J mice were culled by cervical dislocation and muscle excised, snap frozen in liquid nitrogen and stored at -80°C until analysis. All experiments were approved by the University of Auckland Animal Ethics Committee, New Zealand.

### Muscle and blood collection

Following an overnight fast, and having abstained from strenuous physical activity for at least 48h prior to arriving at the laboratory in the morning (before 0900 h), venous blood was drawn into a 10 ml EDTA vacutainer tube, and a percutaneous muscle biopsy was obtained from the vastus lateralis muscle using suction-modified Bergstrom biopsy needle under local anaesthesia. Muscle samples were snap-frozen in liquid nitrogen and blood samples were centrifuged immediately upon collection at 4° C, 1900 x g for 15 min. Plasma and muscle samples were subsequently stored at -80°C until analysis.

### Plasma analysis

Glucose (C311 autoanalyzer, Roche, Mannheim, Germany) and insulin (cobas e11, Roche, Mannheim, Germany) were measured in plasma and the homeostatic model assessment (HOMA) was used to estimate insulin resistance (HOMA2-IR2; The Oxford Centre for Diabetes, Endocrinology and Metabolism, UK) [48]. As described previously [14], MOTS-c was extracted from plasma in acetonitrile-hydrochloric acid and measured using an in-house sandwich ELISA developed at the University of Southern California by the laboratory that first described MOTS-c in human and murine plasma. This ELISA has been extensively validated, and peptide blocking experiments have been undertaken to show specificity of the antibody [14].

### Immunoblotting

~20 mg of muscle was homogenized for 40s at 20 Hz using a TissueLyser (Qiagen, Venlo, Netherlands) in lysis buffer containing 10 μL/mg 25 mM Tris 0.5% v/v Triton X-100 supplemented with Halt Protease and Phosphatase Inhibitor Cocktail (Thermo Scientific, Waltham, MA). The supernatant was collected after centrifugation to determine protein concentration using the BCA protein assay kit (Thermo Scientific, Waltham, MA). Laemmli's buffer was added to protein homogenates and boiled for 5 min at 95 °C. Denatured proteins were separated by SDS-PAGE on 8%–16% TGX pre-cast gels (Bio-Rad, Hercules, USA) and proteins were transferred to PVDF membranes (Bio-Rad, Hong Kong, China) using a TransBlot semidry transfer apparatus (Bio-Rad, Hongkong, China). Membranes were blocked in 5% BSA in TBST for 1h at room temperature, and probed using specific antibodies for MOTS-c (custom produced by YenZym Antibodies, LLC, South San Francisco, CA and described previously [14], 1:500) COXIV (Molecular probes #A6431, 1:10,000) and α-tubulin (Sigma-Aldrich #T9026, 1:10,000). Primary antibodies were incubated overnight at 4 °C with gentle agitation. The following morning, the membranes were probed with either anti-rabbit or anti-mouse linked to horseradish peroxidase secondary antibodies (Jackson ImmunoResearch, West Grove, PA, USA) (1:10,000 dilution) for 1h at room temperature. Membranes were exposed using enhanced chemiluminescence reagent (Western Lightning^®^ Ultra, Perkin Elmer, Waltham, MA, USA) and chemiluminescent signals were captured using ChemiDoc image device (BioRad, HongKong, China). Densitometry analysis of protein bands were quantified using ImageJ software (National Institutes of Health, Bethesda, MD). The intensity of each band was recorded relative to their respective α-tubulin intensity and then normalized to the ratio of target to loading protein in the pooled control sample ran on each gel, and log transformed if required.

### Real-time polymerase chain reaction

Total RNA and DNA was extracted from ~20 mg of tissue using the AllPrep® DNA/RNA/miRNA Universal Kit (QIAGEN GmbH, Hilden, Germany) and small RNA’s (<200 nt) enriched using the Zymo RNA Clean and Concentrator Kit (Cat # R1016, Zymo Research, Irvine, CA) per the manufacturer’s instructions, and enrichment confirmed using RNA NANO Chip using a 2100 Bioanalyzer (Agilent Technologies; data not shown). Total RNA was reverse transcribed using a High-Capacity RNA-to-cDNA™ kit (Life Technologies, Carlsbad, CA) and quantitative real-time PCR was performed on a QuantStudio 6 PCR System using the SYBR green select master mix (Applied Biosystems, Foster City, CA). Reactions were performed in duplicate and relative quantification achieved using the ∆∆Ct method with geometric mean of four reference genes (human sample: *C1ORF43*, *CHMP2A*, *VCP* *EMC7*; mouse sample, *Gapdh, Actb B2mb Rplpo*) as an internal control. Primer sequences used are listed in Supplementary Table 3, and for mitochondrial (mtDNA) to nuclear (nDNA) ratio primers were used to MTND4 and 18S rRNA. For small RNA assays, 50 ng of fractioned RNA was converted to cDNA using TaqMan® microRNA RT kit #4366596 (Life Technologies, Carlsbad, CA) and a multiplexed approach [49] was utilized with specific TaqMan small RNA assays (Life Technologies, Carlsbad, CA) for MOTS-c (designed using Custom TaqMan® Small RNA Assay Design Tool against MOTS-c mtDNA sequence), RNU44 (assay ID: 001094) and RNU48 (assay ID: 001006) primers as per the manufactures instruction and as described previously [50]. The expression of MOTS-c mRNA was normalized to the geomean RNU44 and RNU48 using the ∆∆Ct method.

### Statistical analyses

Statistical analyses were performed using Prism 8 (GraphPad Software Incorporated, California, USA) and SPSS (IBM SPSS, NY, USA, version 25), with statistical significance determined as p ≤ 0.05, and data are presented as individual data points and mean ± standard error of the mean unless stated otherwise. Statistical significance was determined with Pearson linear regression, one-way ANOVA with Holm-Sidak’s post-hoc analysis or ANCOVA.

## Supplementary Material

Supplementary Figures

Supplementary Tables

## References

[1] Flatt T. A new definition of aging? Front Genet. 2012; 3:148. 10.3389/fgene.2012.00148 22936945 PMC3425790

[2] Martin GM, Austad SN, Johnson TE. Genetic analysis of ageing: role of oxidative damage and environmental stresses. Nat Genet. 1996; 13:25–34. 10.1038/ng0596-25 8673100

[3] Jang JY, Blum A, Liu J, Finkel T. The role of mitochondria in aging. J Clin Invest. 2018; 128:3662–70. 10.1172/JCI120842 30059016 PMC6118639

[4] Petersen KF, Befroy D, Dufour S, Dziura J, Ariyan C, Rothman DL, DiPietro L, Cline GW, Shulman GI. Mitochondrial dysfunction in the elderly: possible role in insulin resistance. Science. 2003; 300:1140–42. 10.1126/science.1082889 12750520 PMC3004429

[5] López-Otín C, Blasco MA, Partridge L, Serrano M, Kroemer G. The hallmarks of aging. Cell. 2013; 153:1194–217. 10.1016/j.cell.2013.05.039 23746838 PMC3836174

[6] Shigenaga MK, Hagen TM, Ames BN. Oxidative damage and mitochondrial decay in aging. Proc Natl Acad Sci USA. 1994; 91:10771–78. 10.1073/pnas.91.23.10771 7971961 PMC45108

[7] Merry TL, Ristow M. Mitohormesis in exercise training. Free Radic Biol Med. 2016; 98:123–30. 10.1016/j.freeradbiomed.2015.11.032 26654757

[8] Nakahira K, Haspel JA, Rathinam VA, Lee SJ, Dolinay T, Lam HC, Englert JA, Rabinovitch M, Cernadas M, Kim HP, Fitzgerald KA, Ryter SW, Choi AM. Autophagy proteins regulate innate immune responses by inhibiting the release of mitochondrial DNA mediated by the NALP3 inflammasome. Nat Immunol. 2011; 12:222–30. 10.1038/ni.1980 21151103 PMC3079381

[9] Korolchuk VI, Miwa S, Carroll B, von Zglinicki T. Mitochondria in Cell Senescence: Is Mitophagy the Weakest Link? EBioMedicine. 2017; 21:7–13. 10.1016/j.ebiom.2017.03.020 28330601 PMC5514379

[10] Chandel NS. Evolution of Mitochondria as Signaling Organelles. Cell Metab. 2015; 22:204–06. 10.1016/j.cmet.2015.05.013 26073494

[11] Houtkooper RH, Argmann C, Houten SM, Cantó C, Jeninga EH, Andreux PA, Thomas C, Doenlen R, Schoonjans K, Auwerx J. The metabolic footprint of aging in mice. Sci Rep. 2011; 1:134. 10.1038/srep00134 22355651 PMC3216615

[12] Durieux J, Wolff S, Dillin A. The cell-non-autonomous nature of electron transport chain-mediated longevity. Cell. 2011; 144:79–91. 10.1016/j.cell.2010.12.016 21215371 PMC3062502

[13] Gustafsson CM, Falkenberg M, Larsson NG. Maintenance and Expression of Mammalian Mitochondrial DNA. Annu Rev Biochem. 2016; 85:133–60. 10.1146/annurev-biochem-060815-014402 27023847

[14] Lee C, Zeng J, Drew BG, Sallam T, Martin-Montalvo A, Wan J, Kim SJ, Mehta H, Hevener AL, de Cabo R, Cohen P. The mitochondrial-derived peptide MOTS-c promotes metabolic homeostasis and reduces obesity and insulin resistance. Cell Metab. 2015; 21:443–54. 10.1016/j.cmet.2015.02.009 25738459 PMC4350682

[15] Guo B, Zhai D, Cabezas E, Welsh K, Nouraini S, Satterthwait AC, Reed JC. Humanin peptide suppresses apoptosis by interfering with Bax activation. Nature. 2003; 423:456–61. 10.1038/nature01627 12732850

[16] Cobb LJ, Lee C, Xiao J, Yen K, Wong RG, Nakamura HK, Mehta HH, Gao Q, Ashur C, Huffman DM, Wan J, Muzumdar R, Barzilai N, Cohen P. Naturally occurring mitochondrial-derived peptides are age-dependent regulators of apoptosis, insulin sensitivity, and inflammatory markers. Aging (Albany NY). 2016; 8:796–809. 10.18632/aging.100943 27070352 PMC4925829

[17] Xiao J, Howard L, Wan J, Wiggins E, Vidal A, Cohen P, Freedland SJ. Low circulating levels of the mitochondrial-peptide hormone SHLP2: novel biomarker for prostate cancer risk. Oncotarget. 2017; 8:94900–09. 10.18632/oncotarget.20134 29212276 PMC5706922

[18] Lee C, Yen K, Cohen P. Humanin: a harbinger of mitochondrial-derived peptides? Trends Endocrinol Metab. 2013; 24:222–28. 10.1016/j.tem.2013.01.005 23402768 PMC3641182

[19] Kim SJ, Mehta HH, Wan J, Kuehnemann C, Chen J, Hu JF, Hoffman AR, Cohen P. Mitochondrial peptides modulate mitochondrial function during cellular senescence. Aging (Albany NY). 2018; 10:1239–56. 10.18632/aging.101463 29886458 PMC6046248

[20] Kim SJ, Guerrero N, Wassef G, Xiao J, Mehta HH, Cohen P, Yen K. The mitochondrial-derived peptide humanin activates the ERK1/2, AKT, and STAT3 signaling pathways and has age-dependent signaling differences in the hippocampus. Oncotarget. 2016; 7:46899–912. 10.18632/oncotarget.10380 27384491 PMC5216912

[21] Duan J, Chen Z, Wu Y, Zhu B, Yang L, Yang C. Metabolic remodeling induced by mitokines in heart failure. Aging (Albany NY). 2019; 11:7307–27. 10.18632/aging.102247 31498116 PMC6756899

[22] Kim KH, Son JM, Benayoun BA, Lee C. The Mitochondrial-Encoded Peptide MOTS-c Translocates to the Nucleus to Regulate Nuclear Gene Expression in Response to Metabolic Stress. Cell Metab. 2018; 28:516–524.e7. 10.1016/j.cmet.2018.06.008 29983246 PMC6185997

[23] Ramanjaneya M, Jerobin J, Bettahi I, Bensila M, Aye M, Siveen KS, Sathyapalan T, Skarulis M, Abou-Samra AB, Atkin SL. Lipids and insulin regulate mitochondrial-derived peptide (MOTS-c) in PCOS and healthy subjects. Clin Endocrinol (Oxf). 2019; 91:278–87. 10.1111/cen.14007 31066084

[24] Kim SJ, Miller B, Mehta HH, Xiao J, Wan J, Arpawong TE, Yen K, Cohen P. The mitochondrial-derived peptide MOTS-c is a regulator of plasma metabolites and enhances insulin sensitivity. Physiol Rep. 2019; 7:e14171. 10.14814/phy2.14171 31293078 PMC6640593

[25] Li Q, Lu H, Hu G, Ye Z, Zhai D, Yan Z, Wang L, Xiang A, Lu Z. Earlier changes in mice after D-galactose treatment were improved by mitochondria derived small peptide MOTS-c. Biochem Biophys Res Commun. 2019; 513:439–45. 10.1016/j.bbrc.2019.03.194 30967270

[26] Lu H, Tang S, Xue C, Liu Y, Wang J, Zhang W, Luo W, Chen J. Mitochondrial-Derived Peptide MOTS-c Increases Adipose Thermogenic Activation to Promote Cold Adaptation. Int J Mol Sci. 2019; 20:E2456. 10.3390/ijms20102456 31109005 PMC6567243

[27] Lu H, Wei M, Zhai Y, Li Q, Ye Z, Wang L, Luo W, Chen J, Lu Z. MOTS-c peptide regulates adipose homeostasis to prevent ovariectomy-induced metabolic dysfunction. J Mol Med (Berl). 2019; 97:473–85. 10.1007/s00109-018-01738-w 30725119

[28] Kim SJ, Miller B, Kumagai H, Yen K, Cohen P. MOTS-c: an equal opportunity insulin sensitizer. J Mol Med (Berl). 2019; 97:487–90. 10.1007/s00109-019-01758-0 30788534 PMC6462348

[29] Alis R, Lucia A, Blesa JR, Sanchis-Gomar F. The role of mitochondrial derived peptides (MDPs) in metabolism. J Cell Physiol. 2015; 230:2903–04. 10.1002/jcp.25023 25893266

[30] Fuku N, Pareja-Galeano H, Zempo H, Alis R, Arai Y, Lucia A, Hirose N. The mitochondrial-derived peptide MOTS-c: a player in exceptional longevity? Aging Cell. 2015; 14:921–23. 10.1111/acel.12389 26289118 PMC4693465

[31] Miljkovic N, Lim JY, Miljkovic I, Frontera WR. Aging of skeletal muscle fibers. Ann Rehabil Med. 2015; 39:155–62. 10.5535/arm.2015.39.2.155 25932410 PMC4414960

[32] Ramanjaneya M, Bettahi I, Jerobin J, Chandra P, Abi Khalil C, Skarulis M, Atkin SL, Abou-Samra AB. Mitochondrial-Derived Peptides Are Down Regulated in Diabetes Subjects. Front Endocrinol (Lausanne). 2019; 10:331. 10.3389/fendo.2019.00331 31214116 PMC6554664

[33] Zhuang J, Zhang L, Dai S, Cui L, Guo C, Sloofman L, Yang J. Comparison of multi-tissue aging between human and mouse. Sci Rep. 2019; 9:6220. 10.1038/s41598-019-42485-3 30996271 PMC6470208

[34] Boldrin L, Muntoni F, Morgan JE. Are human and mouse satellite cells really the same? J Histochem Cytochem. 2010; 58:941–55. 10.1369/jhc.2010.956201 20644208 PMC2958137

[35] Flurkey K, Currer JM, Harrison DE. Mouse Models in Aging Research. In: Fox JG, Davisson MT, Quimby FW, Barthold SW, Newcomer CE, Smith AL, eds. The Mouse in Biomedical Research, 2nd Edition: Elsevier). 2007 10.1016/B978-012369454-6/50074-1

[36] Barzilai N, Huffman DM, Muzumdar RH, Bartke A. The critical role of metabolic pathways in aging. Diabetes. 2012; 61:1315–22. 10.2337/db11-1300 22618766 PMC3357299

[37] Du C, Zhang C, Wu W, Liang Y, Wang A, Wu S, Zhao Y, Hou L, Ning Q, Luo X. Circulating MOTS-c levels are decreased in obese male children and adolescents and associated with insulin resistance. Pediatr Diabetes. 2018; 19:1058–64. 10.1111/pedi.12685 29691953

[38] Qin Q, Delrio S, Wan J, Jay Widmer R, Cohen P, Lerman LO, Lerman A. Downregulation of circulating MOTS-c levels in patients with coronary endothelial dysfunction. Int J Cardiol. 2018; 254:23–27. 10.1016/j.ijcard.2017.12.001 29242099

[39] Salminen A, Kaarniranta K, Kauppinen A. Regulation of longevity by FGF21: interaction between energy metabolism and stress responses. Ageing Res Rev. 2017; 37:79–93. 10.1016/j.arr.2017.05.004 28552719

[40] Meex RC, Hoy AJ, Morris A, Brown RD, Lo JC, Burke M, Goode RJ, Kingwell BA, Kraakman MJ, Febbraio MA, Greve JW, Rensen SS, Molloy MP, et al. Fetuin B Is a Secreted Hepatocyte Factor Linking Steatosis to Impaired Glucose Metabolism. Cell Metab. 2015; 22:1078–89. 10.1016/j.cmet.2015.09.023 26603189

[41] Stefan N, Häring HU. The role of hepatokines in metabolism. Nat Rev Endocrinol. 2013; 9:144–52. 10.1038/nrendo.2012.258 23337953

[42] Delezie J, Weihrauch M, Maier G, Tejero R, Ham DJ, Gill JF, Karrer-Cardel B, Rüegg MA, Tabares L, Handschin C. BDNF is a mediator of glycolytic fiber-type specification in mouse skeletal muscle. Proc Natl Acad Sci USA. 2019; 116:16111–20. 10.1073/pnas.1900544116 31320589 PMC6690026

[43] Seldin MM, Peterson JM, Byerly MS, Wei Z, Wong GW. Myonectin (CTRP15), a novel myokine that links skeletal muscle to systemic lipid homeostasis. J Biol Chem. 2012; 287:11968–80. 10.1074/jbc.M111.336834 22351773 PMC3320944

[44] D’Souza AR, Minczuk M. Mitochondrial transcription and translation: overview. Essays Biochem. 2018; 62:309–20. 10.1042/EBC20170102 30030363 PMC6056719

[45] Zeng N, D’Souza RF, Mitchell CJ, Cameron-Smith D. Sestrins are differentially expressed with age in the skeletal muscle of men: A cross-sectional analysis. Exp Gerontol. 2018; 110:23–34. 10.1016/j.exger.2018.05.006 29751091

[46] D’Souza RF, Zeng N, Markworth JF, Figueiredo VC, Hedges CP, Petersen AC, Della Gatta PA, Cameron-Smith D, Mitchell CJ. Whey Protein Supplementation Post Resistance Exercise in Elderly Men Induces Changes in Muscle miRNA’s Compared to Resistance Exercise Alone. Front Nutr. 2019; 6:91. 10.3389/fnut.2019.00091 31249834 PMC6582369

[47] Brzycki M. A Practical Approach to Strength Training. Indianapolis (IN): Blue River; 2012.

[48] Wallace TM, Levy JC, Matthews DR. Use and abuse of HOMA modeling. Diabetes Care. 2004; 27:1487–95. 10.2337/diacare.27.6.1487 15161807

[49] Le Carré J, Lamon S, Léger B. Validation of a multiplex reverse transcription and pre-amplification method using TaqMan(®) MicroRNA assays. Front Genet. 2014; 5:413. 10.3389/fgene.2014.00413 25505484 PMC4244598

[50] Mitchell CJ, D’Souza RF, Schierding W, Zeng N, Ramzan F, O’Sullivan JM, Poppitt SD, Cameron-Smith D. Identification of human skeletal muscle miRNA related to strength by high-throughput sequencing. Physiol Genomics. 2018; 50:416–24. 10.1152/physiolgenomics.00112.2017 29602299

