# Supplementary material for: Increased expression of the mitochondrial derived peptide, MOTS-c, in skeletal muscle of healthy aging men is associated with myofiber composition
Source: Aging (Albany NY). 2020 Mar 17;12(6):5244–58. doi: 10.18632/aging.102944 (PMC7138593; doi:10.18632/aging.102944)
Supplement: Supplementary Figures [file aging-12-102944-s001..pdf]

SUPPLEMENTARY FIGURES

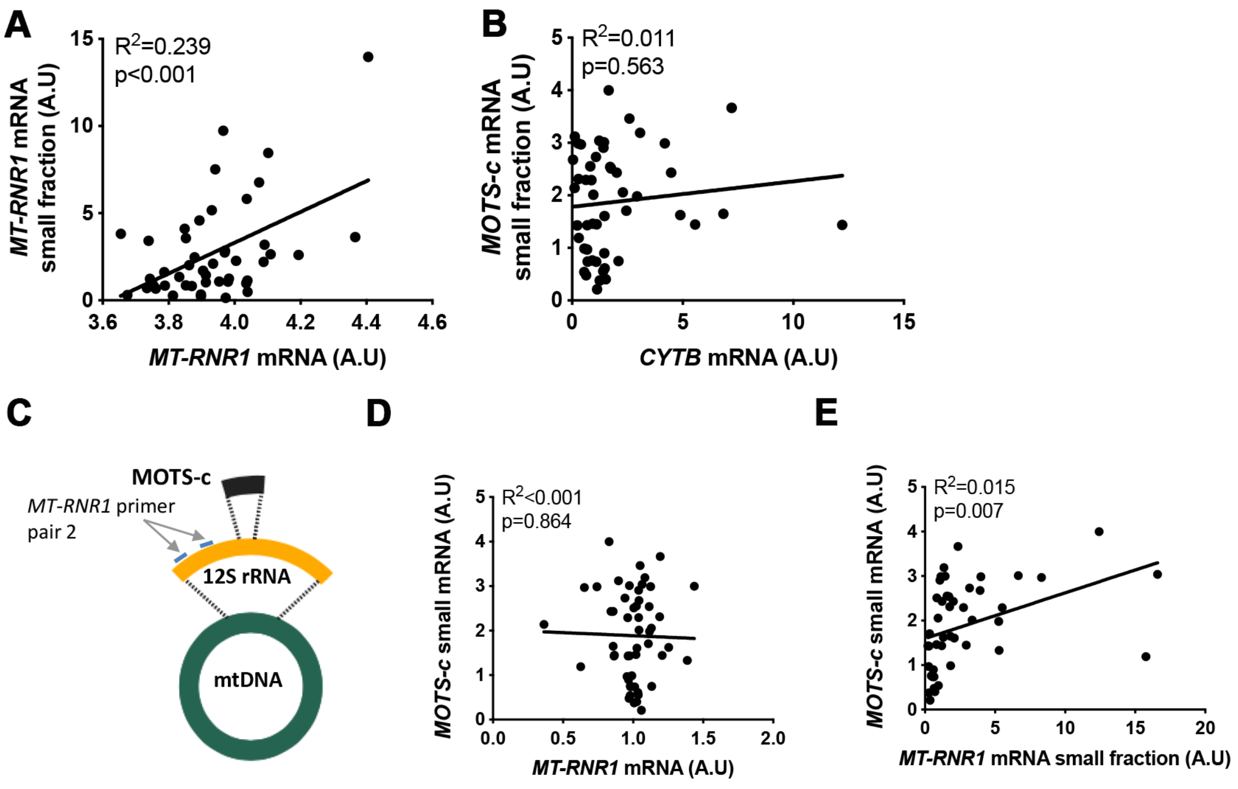

Supplementary Figure 1. Muscle MOTS-c and *12S rRNA* transcription with aging additional data. Correlation between young, middle-aged and older male muscle *12S rRNA mRNA* (*MT-RNR1*) in the total and small mRNA fraction (**A**) and correlation between *MOTS-c* mRNA and *CYTB* mRNA levels in the total RNA fraction (**B**). A second primer pair designed against a different region of *MT-RNR1* (**C**) was used to correlate MOTS-c mRNA levels with *MT-RNR1* mRNA levels in the total (**D**) and small (**E**) RNA fraction.

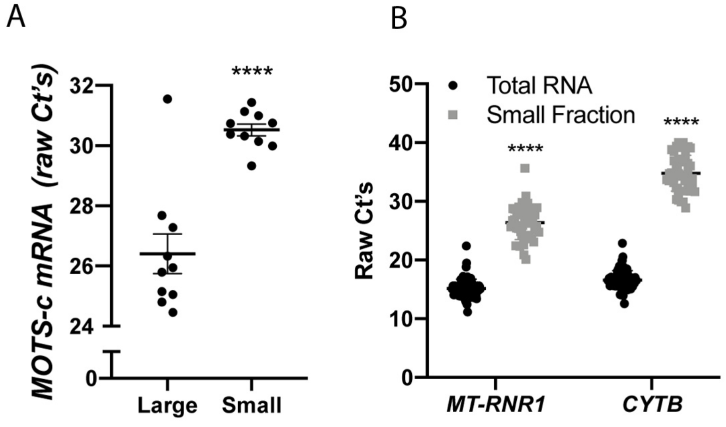

Supplementary Figure 2. Relative gene expression in small and total muscle RNA fractions. Cycle threshold (Ct's) for *MOTS-c* mRNA (**A**) and *MT-RNR1* and *CYTB* (**B**) in RNA fraction enriched for short (<200 nt; small fraction) transcripts or containing longer transcripts (large and total). ****p<0.0001.
