# Supplementary material for: Increased expression of the mitochondrial derived peptide, MOTS-c, in skeletal muscle of healthy aging men is associated with myofiber composition
Source: Aging (Albany NY). 2020 Mar 17;12(6):5244–58. doi: 10.18632/aging.102944 (PMC7138593; doi:10.18632/aging.102944)
Supplement: Supplementary Tables [file aging-12-102944-s002..pdf]

SUPPLEMENTARY TABLES

Supplementary Table 1. ANCOVA analysis of plasma MOTS-c plasma across age groups.

Covariant	F-value	R^2	P-value
None	17.44	0.318	0.000
Lean mass (%)	11.3	0.297	0.000
Fat mass (%)	12.07	0.291	0.000
HOMA-IR	16.31	0.314	0.000
TRIG (mmol/L)	17.61	0.341	0.000
All	11.19	0.334	0.000

Supplementary Table 2. ANCOVA analysis of muscle MOTS-c in young vs. middle group.

Covariant	F-value	R^2	P-value
None	12.09	0.195	0.001
Lean mass (%)	13.64	0.228	0.001
Fat mass (%)	13.85	0.229	0.001
HOMA-IR	11.45	0.205	0.001
TRIG (mmol/L)	12.37	0.210	0.001
All	5.64	0.230	0.022

Supplementary Table 3. Primer sequences.

Gene	Forward primer	Reverse primer
Human DNA		
MT-ND4	CTCTCACTGCCCAAGAACTATC	GGGCTTTAGGGAGTCATAAGTG
18S DNA	CGGAACTGAGGCCATGATTA	ACCTCCGACTTTCGTTCTTG
Human mRNA		
MT-RNR1 (1)	AGCGCAAGTACCCACGTAAA	AGGGCCCTGTTCAACTAAGC
MT-RNR1 (2)	AGTAAGCGCAAGTACCCACG	TAGCCCATTTCTTGCCACCT
C1ORF43	CTATGGGACAGGGGTCTTTGG	TTTGGCTGCTGACTGGTGAT
CHMP2A	CGCTATGTGCGCAAGTTTGT	GGGGCAACTTCAGCTGTCTG
CYTB	TATCCGCCATCCCATACATT	GGTGATTCCTAGGGGGTTGT
EMC7	GGGCTGGACAGACTTTCTAATG	CTCCATTTCCCGTCTCATGTCAG
HMOX1	CAACATCCAGCTCTTTGAGG	GGCAGAATCTTGCACTTTG
MYH2	GCTTTAAAAAGCTCCAAGAACTGTC	ACTTTCGGAGGAAAGGAGCAG
MYH7	AGAAGATGTGCCGGACCTTG	GACAGCTCACCATTCTCGGT
NRF2 (NFE2L2)	GGTTGCCCACATTCCCAAATC	CGTAGCCGAAGAAACCTCA
NQO1	TTGGAGTCCCTGCCATTCTGA	CTGCCTTCTTACTCCGGAAGG
VCP	AAACTCATGGCGAGGTGGAG	TGTCAAAGCGACCAAATCGC
Mouse mRNA		
Myh1	TTATCAAACTGAGGAAGACCGC	CGGAATTTGGCCAGGTTGAC
Myh2	CAAACTGCTGAAGCAGAGGCAAG	CACGAAATGAGGATGGGTGCT
Myh4	TTCCCGAGGCAAACAAGCA	GTTCTTGGCCTTGGACTCTTCCT
Myh7	ACAAAGGCAAAGGCAAGGCAA	GTCCATCACCCCTGGAGACTTTG
Gapdh	CTTTGGCATTGTGGAAGGGC	CAGGGATGATGTTCTGGGCA
Actb	CACTGTCGAGTCGCGTCC	TCATCCATGGCGAACTGGTG
B2mb	CTGCTACGTAACACAGTTCCACCC	CATGATGCTTGATCACATGTCTCG
Rplpo	GGCCCTGCACTCTCGCTTTC	TGCCAGGACGCGCTTGT
